# Irreversible inhibition of BTK kinase by a novel highly selective inhibitor CHMFL-BTK-11 suppresses inflammatory response in rheumatoid arthritis model

**DOI:** 10.1038/s41598-017-00482-4

**Published:** 2017-03-28

**Authors:** Hong Wu, Qiong Huang, Ziping Qi, Yongfei Chen, Aoli Wang, Cheng Chen, Qianmao Liang, Jinghua Wang, Wensheng Chen, Jin Dong, Kailin Yu, Chen Hu, Wenchao Wang, Xiaochuan Liu, Yuanxin Deng, Li Wang, Beilei Wang, Xiaoxiang Li, Nathanael S. Gray, Jing Liu, Wei Wei, Qingsong Liu

**Affiliations:** 1 High Magnetic Field laboratory, Chinese Academy of Sciences, Mailbox 1110, 350 Shushanhu Road, Hefei, 230031 Anhui P.R. China; 2 University of Science and Technology of China, P.R. China, Anhui, Hefei 230036 P.R. China; 3 Institute of Clinical Pharmacology, Anhui Medical University, Key Laboratory of Anti-inflammatory and Immune Medicine, Ministry of Education, Anhui collaborative innovation center of anti-inflammatory and immune medicine, Hefei, 230032 P.R. China; 4 Department of Chemistry, University of Science and Technology of China, Anhui, Hefei 230036 P.R. China; 5 Department of Biological Chemistry & Molecular Pharmacology, Harvard Medical School, 250 Longwood Ave, SGM 628, Boston, MA 02115 USA; 6 Anhui New Star Pharmaceutical Inc., Science Road 110, Hefei, Anhui 230000 P.R. China

## Abstract

BTK plays a critical role in the B cell receptor mediated inflammatory signaling in the rheumatoid arthritis (RA). Through a rational design approach we discovered a highly selective and potent BTK kinase inhibitor (CHMFL-BTK-11) which exerted its inhibitory efficacy through a covalent bond with BTK Cys481. CHMFL-BTK-11 potently blocked the anti-IgM stimulated BCR signaling in the Ramos cell lines and isolated human primary B cells. It significantly inhibited the LPS stimulated TNF-α production in the human PBMC cells but only weakly affecting the normal PBMC cell proliferation. In the adjuvant-induced arthritis rat model, CHMFL-BTK-11 ameliorated the inflammatory response through blockage of proliferation of activated B cells, inhibition of the secretion of the inflammatory factors such as IgG1, IgG2, IgM, IL-6 and PMΦ phagocytosis, stimulation of secretion of IL-10. The high specificity of CHMFL-BTK-11 makes it a useful pharmacological tool to further detect BTK mediated signaling in the pathology of RA.

## Introduction

Rheumatoid arthritis (RA), which is characterized by synovial membrane inflammation and causing joint swelling, cartilage and bone destruction, is an autoimmune inflammation disease that affects about 0.5% of human population^1^. Severe symptoms without effective treatment can result in joint inflammatory destruction that finally leads to disability. A number of cellular responses including chronic activation of T and B lymphocytes mediated innate and adaptive immune cells as well as production of autoantibodies are believed to be important for the pathogenesis of RA. Many of the tyrosine kinases are involved in these processes such as JAK3, Syk, PDGFR, VEGFR, CSF1R, KIT, SRC and BTK etc^2^. Among them, Bruton’s tyrosine kinase (BTK), which is a member of TEC kinase family, is an important downstream mediator after B cell antigen receptor (BCR) activation^3^. Upon SRC kinase family such as Lyn or Syk phosphorylation, BTK will phosphorylate PLCγ2, which will lead to calcium flux and activation of NF-κB and MAPK signaling pathways^4^. Expression of BTK has been found restricted to the B cells but not in T cells or natural killer (NK) cells. As RA is characterized by the B cell activation and subsequent expansion and autoantibody production, BTK has been considered as one of the important potential drug discovery targets for the RA.

Currently a few BTK kinase inhibitors such as GDC-0834^5^ and HM71224^6^ etc are in the clinical development for the RA treatment. Among them GDC-0834 is a reversible BTK kinase inhibitor, while HM71224 is an irreversible inhibitor which exerted its inhibitory efficacy through formation of a covalent bond with cysteine 481, a non-conserved amino acid residue located in the active site of BTK kinase. Here we reported a novel highly selective irreversible BTK kinase inhibitor, CHMFL-BTK-11, which can effectively ameliorate inflammatory response in the adjuvant-induced rodent RA model through modulation of the secretion of the pro/anti-inflammatory factors.

## Results

### Discovery and characterization of CHMFL-BTK-11 as a highly selective and potent BTK kinase inhibitor

**Figure 1:**
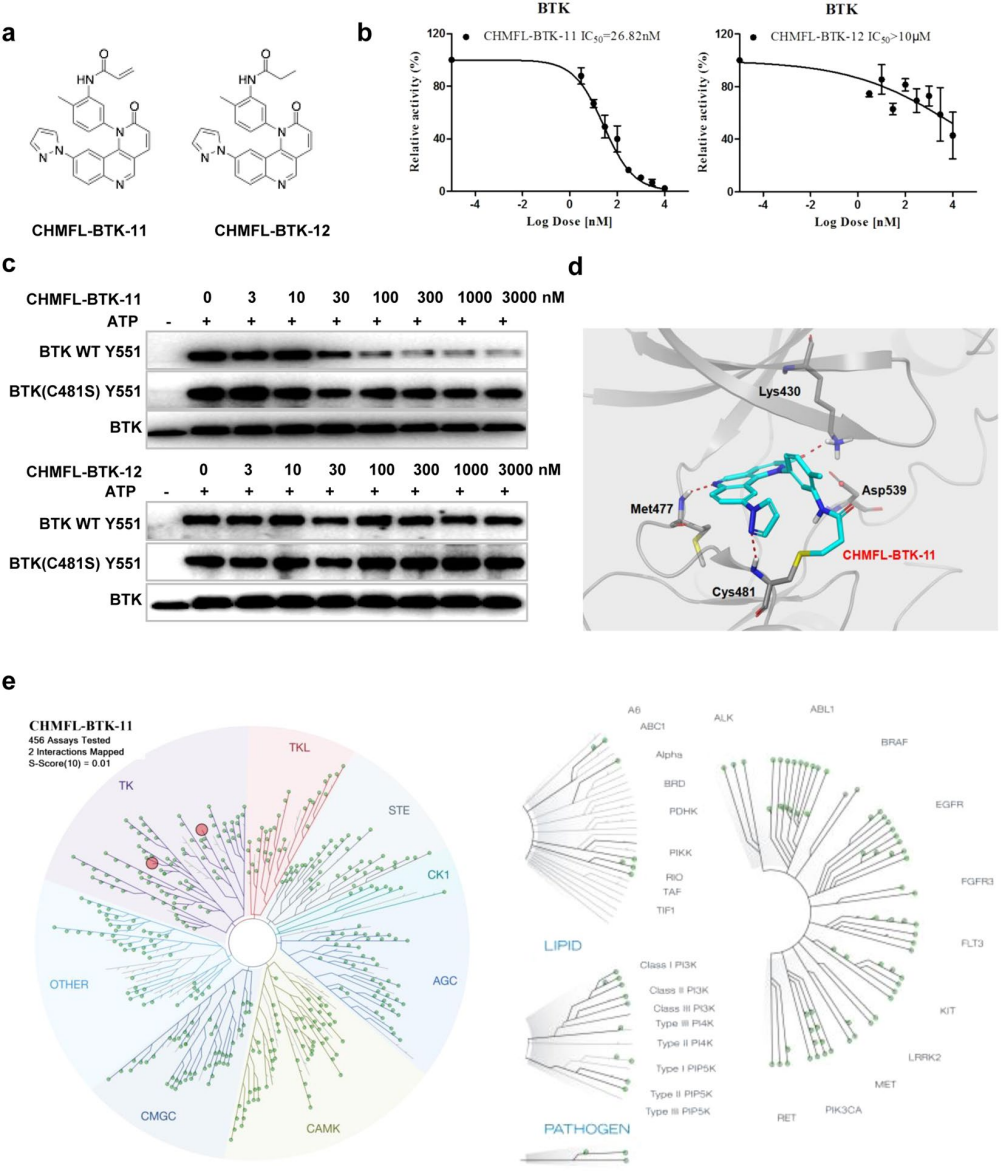
Characterization of CHMFL-BTK-11 as an selective irreversible Bruton’s tyrosine kinase (BTK) inhibitor. (**a**) Chemical structure of CHMFL-BTK-11 and CHMFL-BTK-12; (**b**) ADP-Glo biochemical characterization of CHMFL-BTK-11 and CHMFL-BTK-12 against BTK kinase. (**c**) *In vitro* kinase assay using Flag-tagged BTK of wild-type or C481S immunopurified from HEK293 cells showed that CHMFL-BTK-11 inhibits only wild-type BTK, whereas CHMFL-BTK-12 fails to inhibit both wild-type and C481S BTK. Blots were cropped for improved clarity and conciseness. (**d**) Predicted mode of binding of CHMFL-BTK-11 to BTK based upon molecular modeling (PDB: 3OCS); (**e**) TreeSpot view of the kinase selectivity profile of CHMFL-BTK-11 using data generated from the KinomeScan approach. Data were representative of at least 2 independent experiments.

Starting from a quinoline-based scaffold, by employment of the structure based irreversible inhibitor design approach^7^, we obtained the compound CHMFL-BTK-11 (chemical structure shown in Fig. 1a), which displayed an IC_50_ of 26.82 nM against purified BTK kinase with the ADP-Glo^TM^ biochemical assay (Fig. 1b). While the reversible version compound, CHMFL-BTK-12, in which the nucleophile “warhead” acrylamide was saturated to propionamide, significantly lost the inhibitory activity against BTK (IC_50_: >10 μM) (Fig. 1b). This indicated that CHMFL-BTK-11 might exert its inhibitory efficacy through irreversible binding mode. In order to further confirm the binding mode, we then tested the compounds in the BTK wild-type (wt) and BTK C481S mutant with immunoblotting by looking at the BTK Y551 auto-phosphorylation. The results demonstrated CHMFL-BTK-11 inhibited BTK wt Y551 phosphorylation with an EC_50_ of 25 nM, while BTK C481S was remarkably resistant to it (EC_50_: >3 μM) (Fig. 1c and Supplemental Fig. 1). The reversible version of the compound, CHMFL-BTK-12 did not exhibit apparent inhibitory activity up to 3 μM. Docking CHMFL-BTK-11 into X-ray structure of BTK (PDB ID: 3OCS) showed that a hydrogen bond was formed between the Met-477 and the nitrogen atom in the quinonline. In addition, a covalent bond was preferred to form between the nucleophile acrylamide and Cys-481 near the hinge binding area, which further confirmed its irreversible binding mode (Fig. 1d). We next investigated CHMFL-BTK-11′s selectivity profile in 456 kinases/mutants with KinomeScan^TM^ technology. The results showed that it was highly selective (S Score (10) = 0.01) at the concentration of 1 μM and only strongly bind to BTK kinase and JAK3 kinase (Fig. 1e and Supplemental Table 1). Given the fact the KinomeScan^TM^ is a binding assay and sometimes could not really reflect the compound’s true inhibitory activity, we then tested CHMFL-BTK-11 against purified JAK3 kinase protein with ADP-Glo^TM^ assay which exhibited an IC_50_ of 227 nM. Further testing with TEL fused JAK3 kinase in the engineered BaF3 (TEL-JAK3-BaF3) cells, whose proliferation is dependent on the JAK3 kinase activity, displayed a GI_50_ of 2.3 μM in comparison to the parental BaF3 cells (GI_50_: 4.2 μM), which indicated that in cells, CHMFL-BTK-11 did not potently inhibit JAK3 kinase and it was a highly selective BTK kinase inhibitor.

### CHMFL-BTK-11 inhibits the BTK kinase mediated signaling pathway and affects inflammatory factors secretion

**Figure 2:**
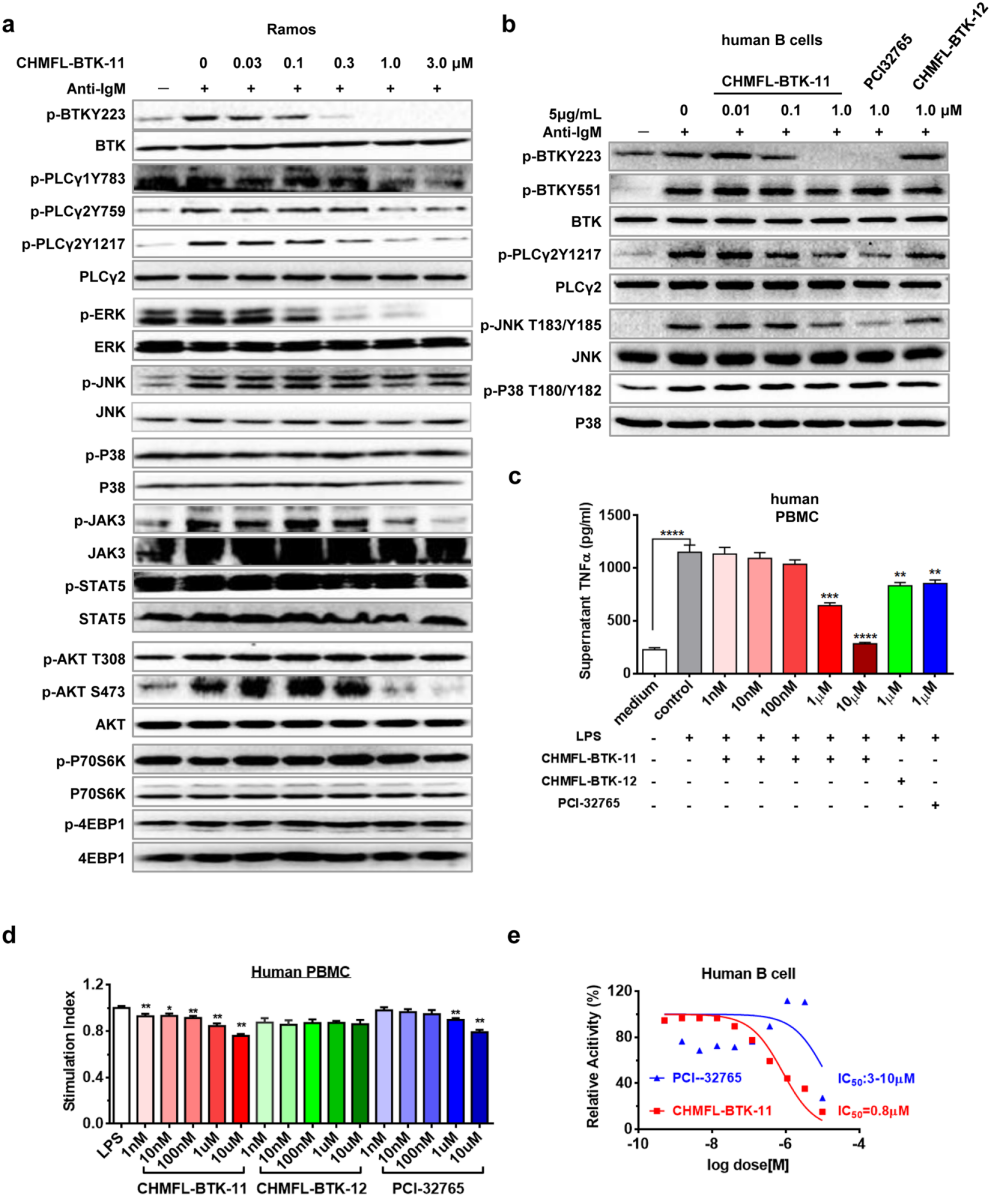
CHMFL-BTK-11 inhibits human B cell activation and proliferation. (**a**) The inhibitory effects of CHMFL-BTK-11 on BTK signaling in the Ramos cell line. Blots were cropped for improved clarity and conciseness. (**b**) The inhibitory effects of CHMFL-BTK-11 on BTK signaling in human B cells. Cells were stimulated with anti-IgM F(ab′)_2_ fragments in the presence of the indicated doses of CHMFL-BTK-11. Blots were cropped for improved clarity and conciseness. (**c**) CHMFL-BTK-11 inhibits TNFα production by human peripheral blood monocytes (PBMC) after LPS stimulation. (**d**) CHMFL-BTK-11 inhibits human PBMC proliferation after LPS stimulation. (**e**) The inhibitory effects of CHMFL-BTK-11 on proliferation of purified human B cells. PCI32765 was set as controls. Student’s t-test was used and data were representative of at least 2 independent experiments. *P < 0.05, **P < 0.01, ***P < 0.001, and ****P < 0.0001. LPS, lipopolysaccharide.

We next investigated CHMFL-BTK-11′s inhibitory efficacy in the Ramos cell line (Burkitt lymphoma cells) which is characterized with high expression of BTK kinase. Upon anti-IgM stimulation, which activates the BTK kinase mediated signaling pathway, CHMFL-BTK-11 potently blocked BTK Y223 phosphorylation (less than 100 nM) (Fig. 2a). It also inhibited downstream mediator PLCγ2 Y1217 phosphorylation (300 nM) but comparably weakly affected PLCγ2 Y759 and PLCγ1 Y783 phosphorylation (1000 nM). ERK phosphorylation was also inhibited at 300 nM and AKT S473 was affected at 1000 nM but AKT T308 was not affected up to 3000 nM. JAK3 phosphorylation was inhibited starting from 1000 nM which further confirmed that CHMFL-BTK-11 is not a highly potent JAK3 inhibitor. Other important inflammatory associated signaling pathway mediators such as STAT5, P38 and JNK were not affected at all. In the purified human B cells, upon anti-IgM stimulation, BTK Y223 and PLCγ2 Y1217 were also potently inhibited by CHMFL-BTK-11 but they were not affected by the reversible version of the compound CHMFL-BTK-12 (Fig. 2b). Given the fact that BTK kinase is also closely associated with TLR4 mediated signaling pathway, we examined the P65 phosphorylation level, which is downstream of TLR4. The results demonstrated that CHMFL-BTK-11 did not affect p-P65 indicating that it might not directly affected TLR4 signaling pathway (Supplemental Fig. 2). The well-established irreversible BTK kinase inhibitor PCI-32765 also exhibited similar effect. These data implied that CHMFL-BTK-11 was a highly potent BTK kinase inhibitor in the cellular background. In the human PBMC cells, upon LPS stimulation, CHMFL-BTK-11 dose-dependently inhibited cytokine TNFα production starting from 1 μM and exhibited better efficacy than reversible version compound CHMFL-BTK-12 and PCI-32765 (Fig. 2c). Meanwhile, CHMFL-BTK-11 only weakly affected PBMC’s proliferation under the same conditions but moderately inhibited the proliferation of normal human B cells (GI_50_: 0.8 μM) (Fig. 2d and e), which indicated that the TNFα production reduction primarily due to the signaling pathway modulation. All the results implied that CHMFL-BTK-11 exerted its efficacy mainly through blocking the BTK mediated signaling in the cellular context.

### CHMFL-BTK-11 reduces the inflammatory responses in the rodent adjuvant-induced arthritis model

**Figure 3:**
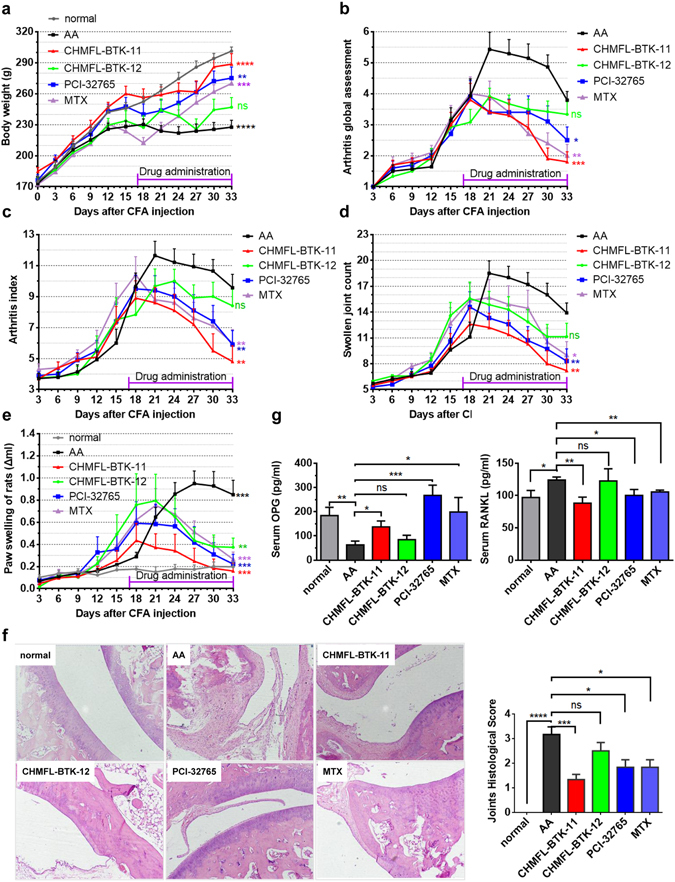
CHMFL-BTK-11 ameliorates symptoms in an adjuvant arthritis (AA) rat model. (**a**–**e**) The body weight (**a**), arthritis global assessment (**b**), arthritis index (**c**), swollen joint count (**d**), and paw swelling of rat (**e**) of AA rats. S.D. rats were intradermal injected with 0.1 ml 10 mg/ml complete Freund’s adjuvant (CFA) into the right rear foot plantar to develop AA rat model. saline injection set as normal control. Drug administration by *i.p*. injection daily begin at day 17, including DMSO (vehicle AA control), 12.5 mg/kg/d CHMFL-BTK-11, 25.0 mg/kg/d CHMFL-BTK-12, 25.0 mg/kg/d PCI-32765, and 0.5 mg/kg/d MTX. (**f**) Histology of ankle joint from AA rats. Data shows HE staining and quantification. (Original magnification 100×). (**g**) Serum OPG and RANKL measurement from AA rats after drug administration. Student’s t-test was used and data were representative of at least 2 independent experiments. ns, P > 0.05,*P < 0.05, **P < 0.01, ***P < 0.001, and ****P < 0.0001.

We then used adjuvant-induced arthritis rat model to evaluate CHMFL-BTK-11′s anti-inflammatory efficacy. The results demonstrated that at 12.5 mg/kg/day dosage, the drug significantly ameliorated rat’s weight loss comparing to the model animal (AA) (Fig. 3a). It displayed better efficacy than 25 mg/kg/day dosage of PCI-32765 and 0.5 mg/kg/every 3 days dosage of Methotrexate (MTX). The reversible version of compound CHMFL-BTK-12 did not exhibit too much efficacy on weights loss. Furthermore, CHMFL-BTK-11 displayed great global anti-arthritis assessment (parameters shown in Supplemental Table 2) efficacy with 12.5 mg/kg/day dosage which was similar to the 0.5 mg/kg/every 3 days MTX and better than 25 mg/kg/day dosage of CHMFL-BTK-12 and PCI-32765 (Fig. 3b). In the arthritis index assessment (parameters shown in Supplemental Table 3), which is another classic indicator in RA, 12.5 mg/kg/day dosage of CHMFL-BTK-11 obtained the best efficacy, while PCI-32765 and MTX treatment generated moderate efficacies, while CHMFL-BTK-12 did not exhibit too much efficacy (Fig. 3c). In the swollen joint counts and paw swelling assessments, 12.5 mg/kg/day of CHMFL-BTK-11 still exhibited the best efficacy, though all other three drugs also reduced swollen joint counts and decreased paw swelling (Fig. 3d,e). Finally, we examined ankle joints inflammatory response by histopathological staining (Fig. 3f and Supplemental Table 4). In AA rats, the joint’s connective tissues were destructed, which showed synovial proliferation, inflammatory cell infiltration. 12.5 mg/kg/day of CHMFL-BTK-11 could protect the rat joints from synovial proliferation, better than 25 mg/kg/day of CHMFL-BTK-12 and PCI-32765 and 0.5 mg/kg/every 3 days of MTX. We also detected OPG and RANKL levels in the serum of AA rats model. As expected, the results demonstrated that upon CHMFL-BTK-11 treatment, level of OPG was increased and RANKL was decreased. And this phenotype was also observed in BTK inhibitor PCI-32765 and canonical anti-inflammatory drug MTX treated groups (Fig. 3g) All the above results indicated that CHMFL-BTK-11 overall exhibited good anti-inflammatory efficacy in the rodent adjuvant-induced arthritis model.

### CHMFL-BTK-11 affects inflammatory factors in the rodent adjuvant-induced arthritis model

**Figure 4:**
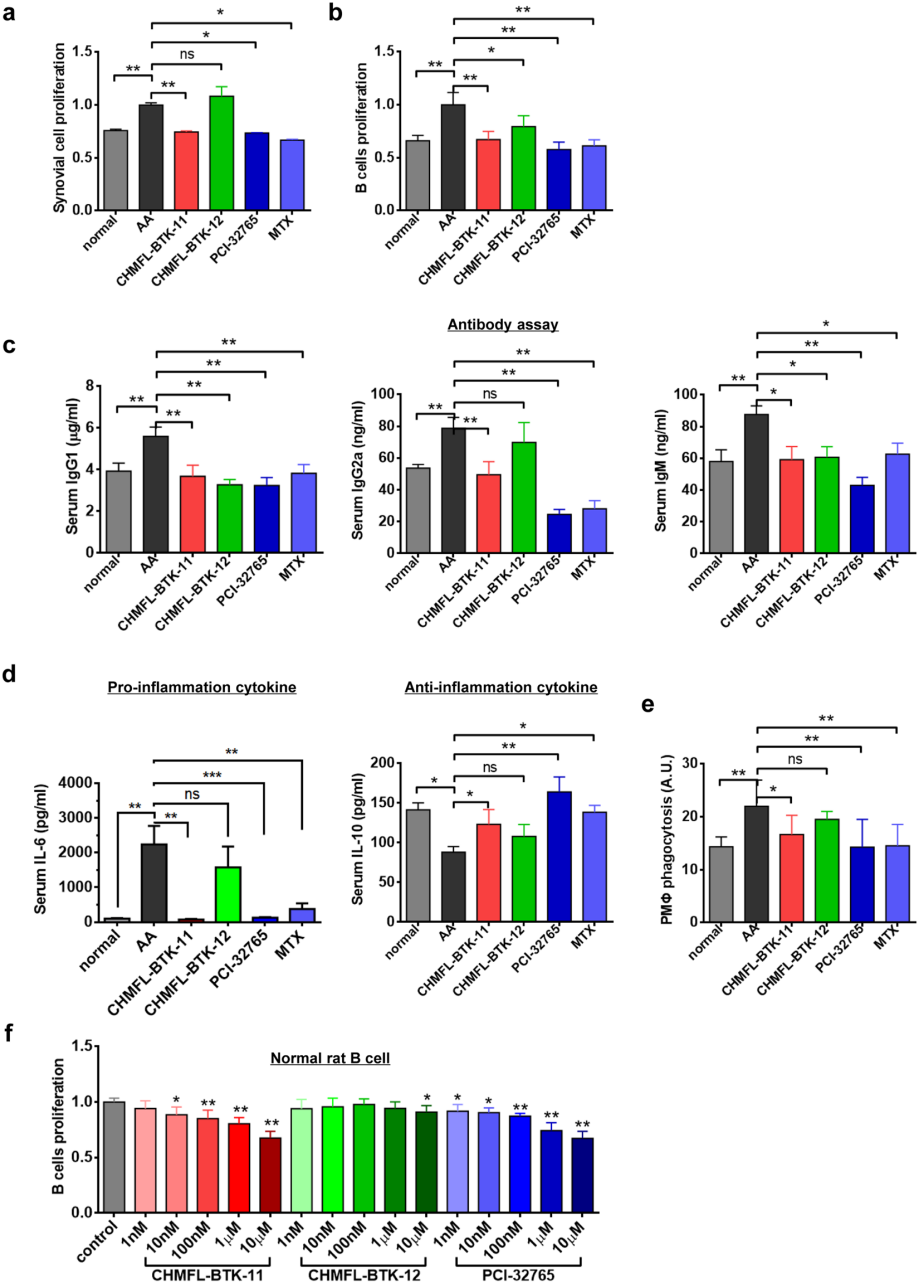
CHMFL-BTK-11 inhibits synovial cells and B cells proliferation and ameliorates total serum antibody production in AA rat model. (**a**) CHMFL-BTK-11 inhibits the synovial cells proliferation from AA rats. (**b**) CHMFL-BTK-11 inhibits B cells proliferation induced by LPS from AA rats. (**c**) Antibodies assay (IgG1, IgG2a, and IgM) from AA rats after drug administration. (**d**) Serum IL-6 and IL-10 measurement from AA rats after drug administration. (**e**) CHMFL-BTK-11 reduced the peritoneal macrophages (PMΦ) phagocytosis from AA rats. (**f**) The inhibitory effects of CHMFL-BTK-11 on proliferation of rat B cells. CHMFL-BTK-12, PCI-32765, or MTX were set as controls. Student’s t-test was used and data were representative of at least 2 independent experiments. ns, P > 0.05, *P < 0.05, **P < 0.01.

In order to understand the mechanism of the CHMFL-BTK-11′s effects for the anti-inflammatory responses in the animal, we collected the synovialis and spleens from animals at the end of the study (day 33) and examined the recall responses *in vitro*. Compared to the enhanced synovial cell proliferation in the AA model, the proliferations in the CHMFL-BTK-11 (12.5 mg/kg/day), PCI-32765 (25 mg/kg/day) and MTX (0.5 mg/kg/3 days) treated animals were all reduced to the normal levels, except for CHMFL-BTK-12, which was still kept in the inflammatory level (Fig. 4a). Similar results were observed for the B cells obtained from rat’s spleen under the LPS stimulation condition (Fig. 4b). Since B cell activation, expansion and autoantibody secretion play important roles in arthritis, we then examined the serum IgG1, IgG2a and IgM autoantibody production in the drug treated AA models (Fig. 4c). The results demonstrated that all drugs could reduce the serum IgG1 and IgM levels to the normal stage. CHMFL-BTK-11 could decrease the serum IgG2a back to the normal level, but not CHMFL-BTK-12. Interestingly, PCI-32765 and MTX reduced the IgG2a to a lever lower than normal. As pro- or anti-inflammation cytokines are often related to the pathology of arthritis, we also examined the secretion levels of IL-1β, IL-6, IL-17A, TNF-α and IL-10 in AA models. Interestingly, CHMFL-BTK-11 (12.5 mg/kg/day) didn’t affect the production of IL-1β, reduced the production of IL-6 but enhanced the production of IL-10. TNF-α and IL-17A was slightly affected (Fig. 4d and Supplemental Fig. 3). In addition, we isolated rat peritoneal macrophages (PMΦ) and compared their phagocytosis after drug treatment. CHMFL-BTK-11 could reduce the excessive PMΦ phagocytosis to normal level in AA rats and similar effects were observed with PCI-32765 and MTX (Fig. 4e). We also isolated B cells from normal rats and tested its proliferation under LPS stimulation upon BTK inhibitors treatment. The results demonstrated that neither CHMFL-BTK-11 nor PCI-32765 had significant anti-proliferation efficacy against activated B-cells (Fig. 4f). In addition, we also examined compound’s anti-proliferative effects on the human (MH7A cell line) and rat’s synovial cells to rule out the possibility of the compound directly affect the synovial cells. The results showed that neither CHMFL-BTK-11 nor PCI-32765 had apparent effects on these cells (Supplemental Fig. 4). All of these data indicated that these BTK inhibitors mainly exerted their anti-inflammatory effect through modulation of the BTK mediated signaling pathways under pathological conditions but not direct killing of the B-cells or synovial cells.

## Discussion

BTK kinase as a therapeutic target for inflammatory disease has attracted more and more attention from the drug discovery community which is envisioned by the several such inhibitors moving to human clinical trials. Among them PCI-32765 and HM71224 are the irreversible inhibitors which utilize cysteine 481 residue located in the region adjacent to hinge binding area. However, there are 11 kinases in the human kinome of over 500 kinases, such as BLK, JAK3, MAP2K7, BMX, BTK, ITK, RLK, TEC, EGFR, Her2, and Her4 kinases, share a similar cysteine residue at the identical sequence position, which makes the inhibitors hard to achieve the selectivity^8^. The first irreversible BTK kinase inhibitor PCI-32765, which has demonstrated the proof-of-concept for the anti-arthritis efficacy by direct inhibition of BTK kinase, also bears potent activities against BLK, BMX, EGFR, Her2, ITK, JAK3, TEC and others^9–11^. HM71224 also potently inhibits BMX, TEC, TXK, EGFR, BLK, ITK, JAK3 kinases besides BTK kinase^6^. While CHMFL-BTK-11 achieved high selectivity among these kinases and the kinome wide selectivity profiling by KinomeScan^TM^ only revealed BTK and JAK3 kinases as the potential targets in the kinome. The biochemical study showed that it exhibits about 10-fold selectivity between BTK and JAK3. Therefore, CHMFL-BTK-11 achieved the best selectivity among the known irreversible BTK kinase inhibitors. The benefits of the high selectivity can be observed by the effects on the serum IgG2 and IgM level regulation. PCI-32765 decreased the IgG2 and IgM to levels lower than normal, which presumably came from the other target modulation and this might result in the increasing possibility of infections during the drug treatment. Given the fact that arthritis is not an acute lethal disease, the safety requirement is usually much more strict than other life-threatening diseases such as cancers. Therefore, a more carefully trimmed selectivity profile of the drug will be more preferred due to the on/off-targets associated toxicity. Furthermore, the role of BTK mediated signaling pathway in the auto-immune diseases is still under extensive study yet and a more selective inhibitor will greatly facilitate the basic mechanistic study than the non-selective inhibitors.

In summary, we have discovered a novel highly selective irreversible BTK kinase inhibitor CHMFL-BTK-11, which could effectively suppress the activation of B cells and the secretions of a variety of cytokines by directly blocking BTK and its downstream signaling cascades. In addition, CHMFL-BTK-11 ameliorated the signs and symptoms of experimental arthritis and prevented ankle joint destruction in the adjuvant-induced rat arthritis model. Furthermore, it only moderately affects B cells proliferation indicating a proper therapeutic safety window. Due to its high selectivity, potency and safety, CHMFL-BTK-11 will be a preferred pharmacological tool for dissection of BTK mediated signaling pathway in the autoimmune diseases and a variety of cancers. Currently CHMFL-BTK-11 is under extensive preclinical safety evaluation for the arthritis.

## Methods

### Inhibitors

CHMFL-BTK-11/12 was synthesized and characterized following the procedure described in the Supplemental Materials. PCI-32765 was purchased from Haoyuan Chemexpress Inc.

### Cell lines and cell culture

Ramos cell line was purchased from the American Type Culture Collection (Manassas, VA, USA). Ramos cells and BaF3 isogenic cell lines cultured in RPMI 1640 (Corning), containing 10% fetal bovine serum (Gibco, Grand Island, NY, USA), 1% penicillin-streptomycin (Gibco, BRL). 293T cells was cultured in DMEM (Corning), containing 10% fetal bovine serum (Gibco, Grand Island, NY, USA), 1% penicillin-streptomycin (Gibco, BRL).

### Antibodies and Immunoblotting

The following antibodies were purchased from Cell Signaling Technology (Danvers, MA): Phospho-Btk (Tyr223) Antibody (#5082), Btk (C82B8) Rabbit mAb (#3533), Phospho-p70 S6 Kinase (Thr389) (108D2) Rabbit mAb (#9234), p70 S6 Kinase (49D7) Rabbit mAb (#2708), Phospho-4E-BP1 (Thr37/46) (236B4) Rabbit mAb (#2855), 4E-BP1 (53H11) Rabbit mAb (#9644), Phospho-PLCγ2 (Tyr1217) Antibody (#3871), Phospho-PLCγ2 (Tyr759) Antibody (#3874), PLCγ2 Antibody (#3872), Phospho-PLCγ1 (Tyr783) Antibody (#2821), Stat5 Antibody(#9363), Phospho-Stat5 (Tyr694) (C71E5) Rabbit mAb(#9314), Akt (pan) (C67E7) Rabbit mAb (#4691), Phospho-Akt (Ser473) (D9E) XP® Rabbit mAb (#4060), Phospho-Akt (Thr308) (C31E5E) Rabbit mAb (#2965), p44/42 MAPK (Erk1/2) (137F5) Rabbit mAb (#4695), Phospho-p44/42 MAPK (Erk1/2) (Thr202/Tyr204) (D13.14.4E) XP® Rabbit mAb(#4370), Phospho-Jak3 (Tyr980/981) (D44E3) Rabbit mAb (#5031), Jak3 (D1H3) Rabbit mAb (#8827), Phospho-p38 MAPK (Thr180/Tyr182) (D3F9) XP® Rabbit mAb (#4511), p38 MAPK Antibody (#9212), Phospho-SAPK/JNK (Thr183/Tyr185) (81E11) Rabbit mAb(#4668), JNK2 (56G8) Rabbit mAb (#9258). Phospho-BTK (Tyr551) Antibody (#441355), Phospho-NF-κBp65(Ser536) (#3033), NF-κB p65 (C22B4) Rabbit mAb (#4764). Antibody was obtained from Invitrogen (Carlsbad, CA). All the antibodies were used at a dilution of 1:1000. Cells were cultured in 10% FBS-containing RPMI. The serially diluted compounds was added to cells for 4 hours then stimulate cells with ani-IgM. The cells were collected and lysed, and cell lysates were analyzed by western blotting.

### BaF3 isogenic cell line generation

Retroviral constructs for BaF3-TEL-JAK3 variants were made based on the pMSCVpuro (Clontech) backbone. For TEL-fusion vectors, the first 1 kb of human TEL gene with an artificial myristoylation sequence (MGCGCSSHPEDD) was cloned into pMSCVpuro retroviral vector, followed by a 3xFLAG tag sequence and a stop codon. Then the kinase domain coding sequences of JAK3 variants were inserted in-frame between TEL and 3xFLAG sequences. For full-length expression vectors, the coding sequences of JAK3 variants were directly cloned in pMSCVpuro vector with a 3xFLAG tag at the C-terminal end. Retrovirus was made using the same method described above and was used to infect BaF3 cells. After puromycin selection, the IL-3 concentration in the medium was gradually withdrawn until cells were able to grow in the absence of IL-3.

### BTK wt/mutant generation

#### Generating BTK stable cell lines

Retroviral expression vectors were constructed by inserting wild-type (wt) and C481S mutant BTK coding regions into a pMSCV-puro vector (Clontech) that was modified by inserting a 3xFLAG sequence at the 3′ end of the multi-cloning sites. Retroviruses of BTK-WT-FLAG and BTK-MT-FLAG were packaged by co-transfecting the plasmids with two helper plasmids into 293T cells using Fugene 6 (Roche). After 48 hours, the medium of 293T cells was replaced with DMEM medium containing 1 µg/ml of puromycin for 48 hours. Then cells were maintained in DMEM medium containing 1 µg/ml of puromycin.

#### Kinase assay

The serially diluted CHMFL-BTK-11 was added to the BTK kinase. After 30 min incubation at RT, ATP (final concentration: 20 μM) was added and incubated for 20 min at 37 °C. The reactions were stopped by addition of 5× sample buffer. Then the phosphorylation of BTK was detected by western blot using phospho-BTK (Tyr551) antibody.

### Human B-cell purification

Fresh human peripheral blood was obtained with agreement from healthy donors. The authors confirm that all methods were carried out in accordance with the written consent under approval of the Chinese Academy of Sciences Institutional Review Board, that all experimental protocols were approved by the Medical Ethics Committee of Hefei Institutes of Physical Science (Chinese Academy of Sciences), and that “informed consent” was obtained from all subjects. After density gradient centrifugation, CD19 ^+^  human B cells were purified using immunomagnetic beads according to the manufacturers’ instructions (Human CD19^+^ B cells selection kit, Miltenyi Biotec, Bergisch-Gladbach, Germany). The purity of CD19^+^ B cells was >95%.

### Anti-proliferation assay

Cells were grown in 96-well culture plates (2500–3000/well) for 12 h before compounds of various concentrations were added. Cell proliferation was determined after treatment with compounds for 72 hours. Cell Titer-Glo assays were performed according to the manufacturer’s instructions; and luminescence was measured in a multi-label reader (Envision, PerkinElmer, USA). Data were normalized to control groups (DMSO) and represented by the mean of three independent measurements with standard error <20%. GI_50_ values were calculated using Prism 5.0 (GraphPad Software, San Diego, CA).

### Animal study

#### Animals

Male Sprague_Dawley (SD) rats [160 ± 20 g, SPF, Certificate no. 2011–002] were housed under standard laboratory condition [temperature (22 ± 2 °C, humidity (50% ± 10%) and a 12-hour light/dark schedule] with free food and water ad libitum. All studies were approved by the Ethics Review Committee for Animal Experimentation of the Institute of Clinical Pharmacology, Anhui Medical University. The authors confirm that all methods were performed in accordance with the relevant guidelines and regulations from the committee mentioned above.

#### Induction and treatment of AA

Arthritis was induced in SD rats via intradermal immunization with 0.1 ml (1 mg/rat) of heat-killed mycobacterium butyricumin liquid paraffin (complete Freund’s adjuvant, CFA) into the right hind metatarsal footpad. The day of CFA immunization was defined as day 0. On day 16, the animals were randomly divided into 6 groups (n = 12 per group), in which AA rats were intraperitoneal injection CHMFL-BTK-11 (12.5 mg/kg/day), CHMFL-BTK-12 (25 mg/kg/day), PCI-32765(25 mg/kg/day), or intragastric administration MTX (0.5 mg/kg, every 3 days) from days 17–33 after immunization. CHMFL-BTK-11, CHMFL-BTK-12 and PCI-32765 (25 mg/kg/day) were dissolved in DMSO and then suspended in normal saline (0.9% NaCl, NS). MTX were suspended in 0.5% sodium carboxymethylcellulose (CMC-Na). In the normal and AA model groups, the rats were given an equal volume of vehicle (NS) at the same time.

#### Experimental observation

AA severity was performed by two independent observers who were blinded to the treatment protocol. All rats were examined the clinical parameters including body weight, arthritis global assessment^12^, arthritis index, swollen joint count and paw swelling degree^13^ every 3 days.

#### Histological examination and Cytokines and antibodies determination

After sacrificed on day 33, the left hind knee joint were dissected fixing in 10% neutral-buffered formalin, then decalcified in 5% formic acid and embedded in paraffin. The sections were stained with hematoxylin and eosin (HE). The ankle joints were histopathologically analyzed for inflammation, synovial proliferation, cellular infiltration, pannus formation, and cartilage erosion (scales 0–4)^14^. Rats’ blood was collected and serum separated, then stored at −80 °C until use. Concentrations of IgG1, IgG2a, IgM, OPG, RANKL, IL-6, IL-1β, IL-17A, TNFα and IL-10 were measured using ELISA kits.

#### Assay of spleen lymphocyte, fibroblast-like synoviocytes (FLSs) proliferation and peritoneal macrophage phagocytic assay

After rats sacrificed, the spleen and synovial tissues from the knees joints were separated. The spleen cells were suspended using lymphocyte separation medium followed 3 times PBS washing. These cells (1 × 10^6^) from each group were suspending in RPMI-1640 (HyClone, Carlsbad, CA, USA) medium then placed in 96-well plates with LPS (4 mg/L). FLSs were harvested from individual tissue using tissue transplantation method and cultured in DMEM supplemented with 20% fetal calf serum, penicillin (200 U/ml), and streptomycin (200 ng/ml). After 3 to 5 passages, the spindle-shaped cells of a homogeneous population of synoviocytes were resuspend at 1 × 10^5^ density in in 96-well plates for 48 h culture. Four hours before the termination of the culture, 10 μl of CCK-8 (Dojindo Laboratories, Kumamoto, Japan) was added followed by 4 h incubation. Then the 450 nm absorbance (A) was measured using an infinite m 1000 pro microplate reader (tecan). The results are presented as the average of triplicate counts.

Peritoneal macrophages (PMs) was collected using cold PBS intra-abdominal injection and centrifuging. The cells were resuspended in RPMI-1640 culture medium with 10% FBS. 1 × 10^6^ cells were placed into 24-well plate and cultured at 37 °C in a 5% CO^2^ humidified incubator for 2 h. After nonadherent cells were removed, PMs were stimulated with LPS (4 mg/L) for 48 h. PMs were added with FITC-dextran in RPMI-1640 culture medium (1 mg/mL) at 37 °C. After 2 h incubation, PMs were digested and washed with cold PBS for triple then were detected by flow cytometry. Negative background control was PMs staining with FITC-dextran at 4 °C for 2 h. The data were shown as mean fluorescence intensities (MFIs).

#### *In vitro* treatment

SD rat was sacrificed and the spleen was separated. The spleen cells were suspended as previous describtion. These cells (1 × 10^6^) were suspending in RPMI-1640 (HyClone, Carlsbad, CA, USA) medium then placed in 96-well plates with LPS (4 mg/L). Different concentration (1 nM, 10 nM, 100 nM, 1 μM, 10 μM) of CHMFL-BTK-11, CHMFL-BTK-12 and PCI-32765 were treated in cells for 48 hours. The B cell proliferation was examined using CCK-8 as previous.

#### Statistical analysis

The two-tailed unpaired Student’s t-test was used for statistical analyses. The experimental data was expressed as mean ± SEM, and the data are representative of at least 2 independent experiments. p < 0.05 was considered statistically significant.

## Electronic supplementary material

CHMFL-BTK-11 supp info
