# Supplementary material for: Irreversible inhibition of BTK kinase by a novel highly selective inhibitor CHMFL-BTK-11 suppresses inflammatory response in rheumatoid arthritis model
Source: Sci Rep. 2017 Mar 28;7:466. doi: 10.1038/s41598-017-00482-4 (PMC5428509; doi:10.1038/s41598-017-00482-4)
Supplement: Supplementary file 1 — CHMFL-BTK-11 supp info [file 41598_2017_482_MOESM1_ESM.pdf]

Supplemental Materials

Irreversible inhibition of BTK kinase by a novel highly selective inhibitor CHMFL-BTK-11 suppresses inflammatory response in rheumatoid arthritis model

Hong Wu[1,2,7], Qiong Huang[3,7], Ziping Qi[1,7], Yongfei Chen[1,7], Aoli Wang[1,2,7], Cheng Chen[1], Qianmao Liang[1,4], Jinghua Wang[5], Wensheng Chen[3], Jin Dong[3], Kailin Yu[1,2], Chen Hu[1,2], Wenchao Wang[1], Xiaochuan Liu[1,4], Yuanxin Deng[1], Li Wang[1,2], Beilei Wang[1,2], Xiaoxiang Li[6], Nathanael S. Gray[5], Jing Liu[1*], Wei Wei[3*], Qingsong Liu[1,2*]

1. High Magnetic Field laboratory, Chinese Academy of Sciences, Mailbox 1110, 350 Shushanhu Road, Hefei 230031, Anhui, P. R. China
2. University of Science and Technology of China, P. R. China, Anhui, Hefei, 230036, P. R. China
3. Institute of Clinical Pharmacology, Anhui Medical University, Key Laboratory of Anti-inflammatory and Immune Medicine, Ministry of Education, Anhui collaborative innovation center of anti-inflammatory and immune medicine, Hefei, 230032， P.R. China
4. Department of Chemistry, University of Science and Technology of China, Anhui, Hefei, 230036, P. R. China
5. Department of Biological Chemistry & Molecular Pharmacology, Harvard Medical School, 250 Longwood Ave, SGM 628, Boston, MA 02115, USA.
6. Anhui New Star Pharmaceutical Inc. Science Road 110, Hefei, Anhui, 230000, P. R. China
7. These authors contribute equally

Correspondence:

Jing Liu, Ph.D, Email: jingliu@hmfl.ac.cn
Wei Wei, Ph.D, Email: wwei@ahmu.edu.cn
Qingsong Liu, Ph.D, Email: qsliu97@hmfl.ac.cn

Table of Contents:

Chemical synthesis and characterization of CHMFL-BTK-11

Supplemental Figure 1: Quantification of CHMFL-BTK-11/12 effect on the in vitro BTK wt/C481S mutant assay.

Supplemental Figure 2: CHMFL-BTK-11/12 and PCI-32765 effect on the phospho-P65 in the anti-IgM stimulated human PBMC.

Supplemental Figure 3: CHMFL-BTK-11/12, PCI-32765 and MTX effects on the secretion of IL-1β, IL-17A and TNF-α.

Supplemental Figure 4: CHMFL-BTK-11/12, PCI-32765 anti-proliferative effects on the human MH7A cell line and rat arthritis synovial cells.

Supplemental Materials

Supplemental Table 1: Kinome wide selectivity profiling of CHMFL-BTK-11

Supplemental Table 2: The standards of arthritis global assessment* of AA rats

Supplemental Table 3: The evaluation standards of arthritis index* of AA rats

Supplemental Table 4: The evaluation standards of quantification of H&E staining

Chemical synthesis and characterization of CYF-2-11 (CHMFL-BTK-11)

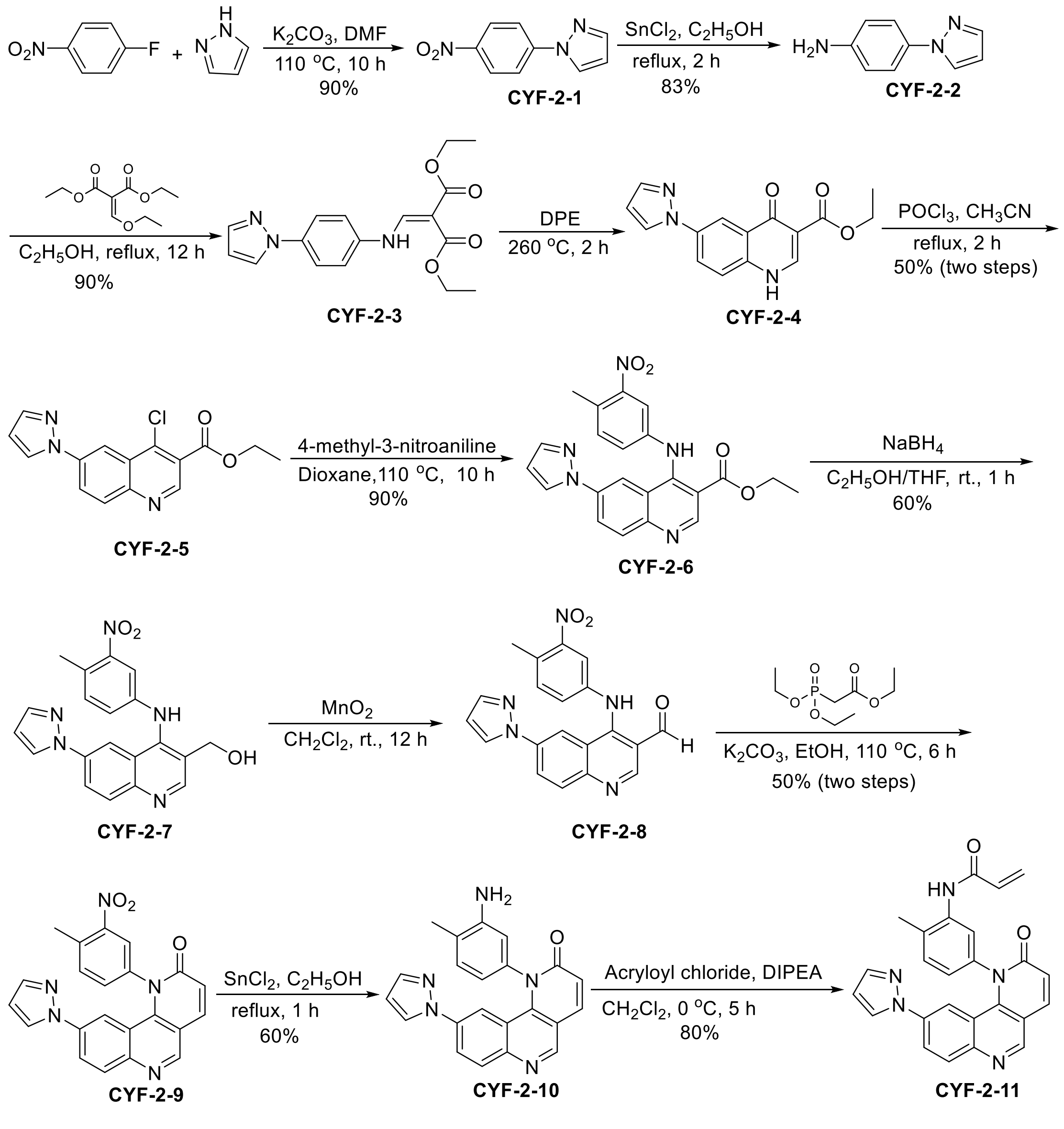

1-(4-nitrophenyl)-1*H*-pyrazole (CYF-2-1)

A mixture of pyrazole (12 mmol, 0.82 g), 4-fluoro-1-nitrobenzene (10 mmol, 1.41 g), potassium carbonate (15 mmol, 2.07 g) in N,N-dimethylformamide (20 mL) was heated

at 110 ℃ for 10 hours and then cooled to room temperature. The reaction mixture was poured into water (100ml) and filtered. The filter cake was washed with water to give 1-(4-nitrophenyl)-1*H*-pyrazole (CYF-2-1) (1.70 g, 90% yield) as pale yellow crystals. ^{1}H-NMR (400 MHz, DMSO) *δ* 8.75 (s, 1H), 8.36 (d, *J* = 4.0 Hz, 2H), 8.14 (d, *J* = 4.4 Hz, 2H), 7.90 (s, 1H), 6.67 (s, 1H); MS (ESI): m/z $(M+H)^+$ 190.0614.

4-(1*H*-pyrazol-1-yl)aniline (CYF-2-2)

$SnCl_2.2H_2O$ (13.1g, 50 mmol) was added to a solution of 4-(3-nitrophenyl)-l*H*-pyrazole (0.95 g, 5 mmol) in EtOH (50 mL). The reaction mixture was refluxed for 2 hours and then cooled to room temperature. The reaction mixture was was neutralized with 1 N NaOH to pH = 8 and extracted with EtOAc. The combined organic layers were washed with brine, dried over anhydrous Na_2S0_4, and concentrated to afford the crude product **CYF-2-2** (0.66 g, 83% yield). MS (ESI): m/z $(M+H)^+$ 160.0877.

Diethyl 2-(((4-(1*H*-pyrazol-1-yl)phenyl)amino)methylene)malonate (CYF-2-3)

A mixture of 1-phenyl-5-aminopyrazole (0.64 g, 4 mmol) and diethyl ethoxymethylenemalonate (1.3 g, 6 mmol) was refluxed in EtOH (15 mL) for 12 hours. After cooling, the reaction mixture was filtered to afford the desired product **CYF-2-3** (1.20 g, 90% yield). ^{1}H-NMR (400 MHz, DMSO) *δ* 10.78 (d, *J* = 13.6 Hz, 1H), 8.50 (s, 1H), 8.43 (d, *J* = 13.2 Hz, 1H), 7.85 (s, 2H), 7.75 (s, 1H), 7.52 (d, *J* = 4.0 Hz, 2H), 6.54 (s, 1H), 4.22 (s, 2H), 4.13 (s, 1H), 1.26 (s, 6H); MS (ESI): m/z $(M+H)^+$ 330.1450.

Ethyl 4-chloro-6-(1*H*-pyrazol-1-yl)quinoline-3-carboxylate (CYF-2-5)

CYF-2-3 (1.00 g, 3 mmol) was added to diphenyl ether (30 mL) and the reaction mixture heated to 260 ℃ for 2 h. After cooling, the corresponding solid was isolated as a gray−white solid by filtration and washing with cold petroleum. The resultant solid (0.50 g, 1.8 mmol) and $POCl_3$ (0.55 g, 3.6 mmol) in MeCN (2 mL) was refluxed for 2 h. Excess $POCl_3$ was removed under reduced pressure and the crude residue was neutralized with saturated sodium bicarbonate solution (100 mL) and extracted with ethyl acetate. The organic layer was dried over Na_2SO_4, filtered and evaporated under reduced pressure. The residue was purified by column chromatography to afford the desired product **CYF-2-5** (0.45 g, 50% yield, two steps). ^{1}H-NMR (400 MHz, DMSO) *δ* 9.14 (d, *J* = 1.6 Hz,

1H), 8.86 (d, *J* = 2.4 Hz, 1H), 8.74 (d, *J* = 2.0 Hz, 1H), 8.56 (dd, *J* = 9.6, 2.4 Hz, 1H), 8.30 (d, *J* = 9.6 Hz, 1H), 7.91 (s, 1H), 6.68 (d, *J* = 2.0 Hz, 1H), 4.45 (q, *J* = 7.2 Hz, 2H), 1.40 (d, *J* = 7.2 Hz, 3H); MS (ESI): m/z $(M+H)^+$ 302.0698.

Ethyl 4-((4-methyl-3-nitrophenyl)amino)-6-(1*H*-pyrazol-1-yl)quinoline-3-carboxy late (CYF-2-6)

To a solution of **CYF-2-5** (0.30 g, 1 mmol) in 1,4-dioxane (3 mL) at room temperature in a sealed tube was added 4-methyl-3-nitroaniline (0.18 g, 1.2 mmol). The resultant solution was heated to 110 ^oC for 10 h. After the mixture was cooled to room temperature, a solution of NaOH (1N) was added to neutralize the solution followed by dilution with water and extraction with ethyl acetate. After the organic extracts were dried over Na_2SO_4, the solvents were removed and the residue was purified by flash column chromatography to furnish product **CYF-2-6** (0.37 g, 90% yield). ^1H-NMR (400 MHz, DMSO) δ 9.75 (s, 1H), 8.89 (s, 1H), 8.70 (d, *J* = 2.4 Hz, 1H), 8.60 (d, *J* = 2.4 Hz, 1H), 8.37 (dd, *J* = 8.8, 2.0 Hz, 1H), 8.13 (d, *J* = 9.2 Hz, 1H), 7.84 (d, *J* = 1.6 Hz, 1H), 8.68 (d, *J* = 2.0 Hz, 1H), 7.41 (d, *J* = 8.4 Hz, 1H), 7.28 (dd, *J* = 8.0, 2.0 Hz, 1H), 6.64 (d, *J* = 1.6 Hz, 1H), 3.35 (q, *J* = 6.8 Hz, 2H), 2.47 (s, 3H), 1.08 (t, *J* = 6.8 Hz, 3H); MS (ESI): m/z $(M+H)^+$ 418.1520.

(4-((4-methyl-3-nitrophenyl)amino)-6-(1*H*-pyrazol-1-yl)quinolin-3-yl)methanol (CYF-2-7)

To a solution of compound **CYF-2-6** (0.33 g, 0.8 mmol) in EtOH/THF (1 mL/3 mL) at 0 ^oC was added $NaBH_4$ (0.6g, 16 mmol). The resultant solution was stirred at room temperature for 1 h before being diluted with EtOAc and filtered through Celite. The filtrate was concentrated under vacuum, and the residue was dissolved in EtOAc and brine. After separation, organic layer was dried over $MgSO_4$ and concentrated under vacuum. The residue was purified by flash column chromatography to afford product **CYF-2-7** (0.18 g, 60% yield). ^1H-NMR (400 MHz, DMSO) δ 9.01 (s, 1H), 8.90 (s, 1H), 8.60 (s, 1H), 8.45 (s, 1H), 8.28 (d, *J* = 8.8 Hz, 1H), 8.17 (d, *J* = 9.2 Hz, 1H), 7.80 (d, *J* = 1.2 Hz, 1H), 7.32 (s, 1H), 7.30 (d, *J* = 9.2 Hz, 1H), 6.91 (d, *J* = 8.0 Hz, 1H), 6.60 (d, *J* =

1.6 Hz, 1H), 5.44 (m, 1H), 4.50 (d, *J* = 5.2 Hz, 2H), 2.41 (s, 3H); MS (ESI): m/z $(M+H)^+$ 376.1404.

1-(4-methyl-3-nitrophenyl)-9-(1*H*-pyrazol-1-yl)benzo[*h*][1,6]naphthyridin-2(1*H*)-one (CYF-2-9)

To a solution of compound **CYF-2-7** (187 mg, 0.5 mmol) in CH_2Cl_2 (5 mL) at room temperature was added MnO_2 (260 mg, 3 mmol). After 12 h, the reaction mixture was filtered through Celite. The filtrate was concentrated in a sealed tube and dissolved in dry EtOH (5 mL), after which K_2CO_3 (410 mg, 3 mmol) and triethyl phosphonoacetate (81 mg, 0.36 mmol) were added. The resulting mixture was heated to 110 ^oC for 6 h before cooling to room temperature. Upon removal of the solvents under vacuum, the residue was diluted with water followed by extraction with EtOAc. The residue was purified by flash column chromatography to provided product **CYF-2-9** (100 mg, 50% yield, two steps). ^{1}H-NMR (400 MHz, DMSO) δ 9.16 (s, 1H), 8.35 (d, *J* = 9.2 Hz, 1H), 8.34 (d, *J* = 1.6 Hz, 1H), 8.22 - 8.11 (m, 3H), 8.00 – 7.73 (d, *J* = 9.2 Hz, 2H), 7.64 (s, 1H), 7.28 (d, *J* = 2.0 Hz, 1H), 6.99 (d, *J* = 9.2 Hz, 1H), 6.53 (dd, *J* = 2.4, 2.0 Hz, 1H), 2.71 (s, 3H); MS (ESI): m/z $(M+H)^+$ 398.1257.

1-(3-amino-4-methylphenyl)-9-(1*H*-pyrazol-1-yl)benzo[*h*][1,6]naphthyridin-2 (1*H*)-one (CYF-2-10)

To a solution of compound **CYF-2-9** (40 mg, 0.1 mmol) in EtOH (2 ml) at room temperature, $SnCl_2 .2H_2O$ (226 mg, 1 mmol) was added. The resultant solution was refluxed for 1 h. After the mixture was cooled to room temperature, a solution of saturated sodium bicarbonate was added to quench the reaction followed by dilution with water and extraction with ethyl acetate. After the organic extracts were dried over Na_2SO_4, the solvents were removed to afford the crude product **CYF-2-10** (24 mg, 60% yield); MS (ESI): m/z $(M+H)^+$ 368.1514.

N-(2-methyl-5-(2-oxo-9-(1*H*-pyrazol-1-yl)benzo[*h*][1,6]naphthyridin-1(2*H*)-yl) phenyl) acrylamide (CYF-2-11/CHMFL-BTK-11)

Supplemental Materials

The crude compound **CYF-2-10** (7.3 mg, 0.02 mmol) was dissolved in dry CH_2Cl_2 (0.4 mL) in an ice bath. Diisopropylethylamine (28 mg, 0.06 mmol) was added. After 5 min, acryloyl chloride (3 mg, 0.03 mmol) was added, and the reaction mixture was stirred for additional 5 h. The solvent was evaporated, and the residue was dissolved in ethyl acetate and washed three times with a $NaHCO_3$ solution (4%). The organic phase was dried over Na_2SO_4, filtered, and concentrated. The residue was purified by flash column chromatography to afford product **CYF-2-11/CHMFL-BTK-11** (7.0 g, 80% yield). ^{1}H-NMR (400 MHz, DMSO) δ 9.79(s, 1H), 9.14(s, 1H), 8.32 (d, *J* = 9.6 HZ, 1H), 8.20 (dd, *J* = 8.8, 2.0 HZ, 1H), 8.15 (d, *J* = 9.2 HZ, 1H), 7.71-7.69 (m, 2H), 7.63 (s, 1H), 7.54 (d, *J* = 8.4 HZ, 1H), 7.24 (d, *J* = 7.6 HZ, 1H), 7.13 (s, 1H), 6.95 (d, *J* = 9.2 HZ, 1H), 6.57-6.50 (m, 2H), 6.20 (d, *J* = 17.2 HZ, 1H), 5.76 (d, *J* = 10.8 HZ, 1H), 2.42 (s, 3H); MS (ESI): m/z $(M+H)^+$ 422.1621.

Supplemental Figure 1: Quantification of CHMFL-BTK-11/12 effect on the in vitro BTK wt/C481S mutant assay

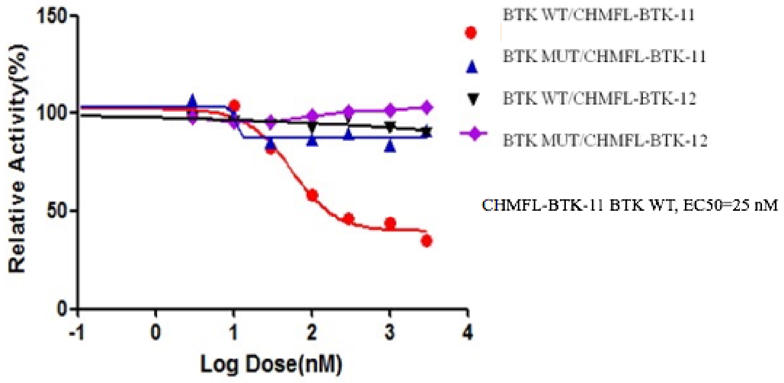

Supplemental Figure 2: CHMFL-BTK-11/12 and PCI-32765 effect on the phospho-P65 in the anti-IgM stimulated human PBMC.

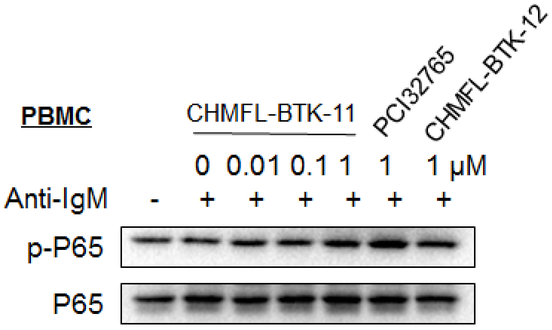

Supplemental Figure 3: CHMFL-BTK-11/12, PCI-32765 and MTX effects on the secretion of IL-1β, IL-17A and TNF-α.

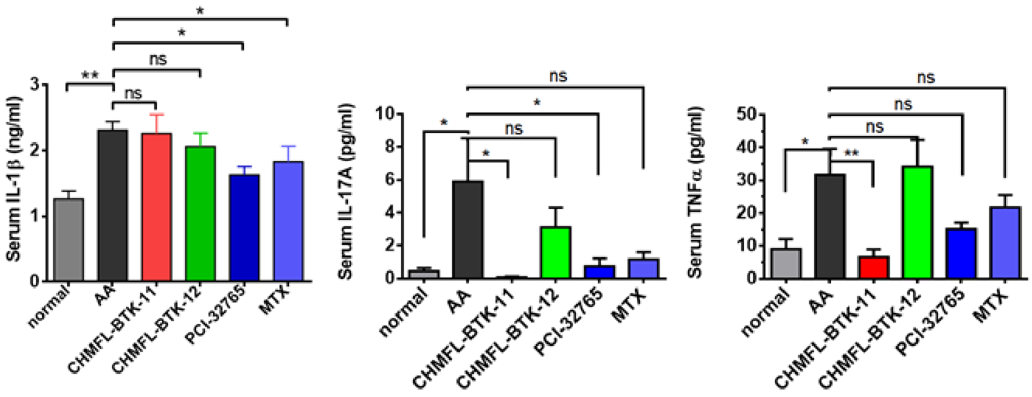

Supplemental Figure 4: CHMFL-BTK-11/12, PCI-32765 anti-proliferative effects on the human MH7A cell line and rat arthritis synovial cells.

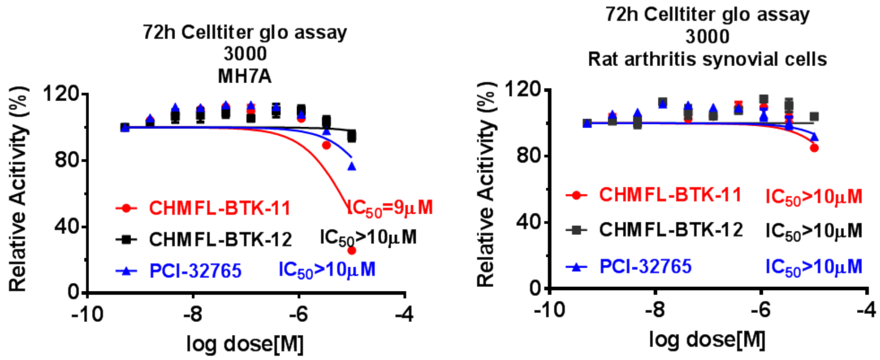

Supplemental Table 1: Kinome wide selectivity profiling of CHMFL-BTK-11

**Compound Name**	**DiscoveRx Gene Symbol**	**Percent Control**	**Compound Concentration (nM)**
CHMFL-BTK-11	AAK1	62	1000
CHMFL-BTK-11	ABL1(E255K)-phosphorylated	92	1000
CHMFL-BTK-11	ABL1(F317I)-nonphosphorylated	100	1000
CHMFL-BTK-11	ABL1(F317I)-phosphorylated	100	1000
CHMFL-BTK-11	ABL1(F317L)-nonphosphorylated	96	1000
CHMFL-BTK-11	ABL1(F317L)-phosphorylated	100	1000
CHMFL-BTK-11	ABL1(H396P)-nonphosphorylated	100	1000
CHMFL-BTK-11	ABL1(H396P)-phosphorylated	100	1000
CHMFL-BTK-11	ABL1(M351T)-phosphorylated	72	1000
CHMFL-BTK-11	ABL1(Q252H)-nonphosphorylated	100	1000
CHMFL-BTK-11	ABL1(Q252H)-phosphorylated	100	1000
CHMFL-BTK-11	ABL1(T315I)-nonphosphorylated	79	1000
CHMFL-BTK-11	ABL1(T315I)-phosphorylated	100	1000
CHMFL-BTK-11	ABL1(Y253F)-phosphorylated	89	1000
CHMFL-BTK-11	ABL1-nonphosphorylated	74	1000
CHMFL-BTK-11	ABL1-phosphorylated	78	1000
CHMFL-BTK-11	ABL2	100	1000

Supplemental Materials

CHMFL-BTK-11	ACVR1	100	1000
CHMFL-BTK-11	ACVR1B	100	1000
CHMFL-BTK-11	ACVR2A	100	1000
CHMFL-BTK-11	ACVR2B	95	1000
CHMFL-BTK-11	ACVRL1	90	1000
CHMFL-BTK-11	ADCK3	96	1000
CHMFL-BTK-11	ADCK4	100	1000
CHMFL-BTK-11	AKT1	96	1000
CHMFL-BTK-11	AKT2	100	1000
CHMFL-BTK-11	AKT3	100	1000
CHMFL-BTK-11	ALK	100	1000
CHMFL-BTK-11	ALK(C1156Y)	94	1000
CHMFL-BTK-11	ALK(L1196M)	88	1000
CHMFL-BTK-11	AMPK-alpha1	100	1000
CHMFL-BTK-11	AMPK-alpha2	86	1000
CHMFL-BTK-11	ANKK1	81	1000
CHMFL-BTK-11	ARK5	100	1000
CHMFL-BTK-11	ASK1	90	1000
CHMFL-BTK-11	ASK2	100	1000
CHMFL-BTK-11	AURKA	71	1000
CHMFL-BTK-11	AURKB	94	1000
CHMFL-BTK-11	AURKC	98	1000
CHMFL-BTK-11	AXL	100	1000
CHMFL-BTK-11	BIKE	91	1000
CHMFL-BTK-11	BLK	30	1000
CHMFL-BTK-11	BMPR1A	100	1000
CHMFL-BTK-11	BMPR1B	97	1000
CHMFL-BTK-11	BMPR2	100	1000
CHMFL-BTK-11	BMX	23	1000
CHMFL-BTK-11	BRAF	93	1000
CHMFL-BTK-11	BRAF(V600E)	100	1000
CHMFL-BTK-11	BRK	100	1000
CHMFL-BTK-11	BRSK1	100	1000
CHMFL-BTK-11	BRSK2	86	1000
CHMFL-BTK-11	BTK	8.2	1000
CHMFL-BTK-11	BUB1	83	1000
CHMFL-BTK-11	CAMK1	98	1000
CHMFL-BTK-11	CAMK1D	100	1000
CHMFL-BTK-11	CAMK1G	84	1000
CHMFL-BTK-11	CAMK2A	100	1000
CHMFL-BTK-11	CAMK2B	100	1000
CHMFL-BTK-11	CAMK2D	80	1000
CHMFL-BTK-11	CAMK2G	73	1000
CHMFL-BTK-11	CAMK4	91	1000
CHMFL-BTK-11	CAMKK1	78	1000
CHMFL-BTK-11	CAMKK2	78	1000
CHMFL-BTK-11	CASK	86	1000
CHMFL-BTK-11	CDC2L1	100	1000
CHMFL-BTK-11	CDC2L2	100	1000
CHMFL-BTK-11	CDC2L5	98	1000
CHMFL-BTK-11	CDK11	89	1000
CHMFL-BTK-11	CDK2	100	1000
CHMFL-BTK-11	CDK3	100	1000
CHMFL-BTK-11	CDK4-cyclinD1	97	1000
CHMFL-BTK-11	CDK4-cyclinD3	100	1000
CHMFL-BTK-11	CDK5	100	1000
CHMFL-BTK-11	CDK7	89	1000

CHMFL-BTK-11	CDK8	91	1000
CHMFL-BTK-11	CDK9	96	1000
CHMFL-BTK-11	CDKL1	93	1000
CHMFL-BTK-11	CDKL2	81	1000
CHMFL-BTK-11	CDKL3	90	1000
CHMFL-BTK-11	CDKL5	100	1000
CHMFL-BTK-11	CHEK1	100	1000
CHMFL-BTK-11	CHEK2	100	1000
CHMFL-BTK-11	CIT	98	1000
CHMFL-BTK-11	CLK1	92	1000
CHMFL-BTK-11	CLK2	100	1000
CHMFL-BTK-11	CLK3	100	1000
CHMFL-BTK-11	CLK4	90	1000
CHMFL-BTK-11	CSF1R	91	1000
CHMFL-BTK-11	CSF1R-autoinhibited	70	1000
CHMFL-BTK-11	CSK	100	1000
CHMFL-BTK-11	CSNK1A1	100	1000
CHMFL-BTK-11	CSNK1A1L	100	1000
CHMFL-BTK-11	CSNK1D	100	1000
CHMFL-BTK-11	CSNK1E	100	1000
CHMFL-BTK-11	CSNK1G1	100	1000
CHMFL-BTK-11	CSNK1G2	100	1000
CHMFL-BTK-11	CSNK1G3	100	1000
CHMFL-BTK-11	CSNK2A1	92	1000
CHMFL-BTK-11	CSNK2A2	52	1000
CHMFL-BTK-11	CTK	65	1000
CHMFL-BTK-11	DAPK1	100	1000
CHMFL-BTK-11	DAPK2	100	1000
CHMFL-BTK-11	DAPK3	100	1000
CHMFL-BTK-11	DCAMKL1	91	1000
CHMFL-BTK-11	DCAMKL2	100	1000
CHMFL-BTK-11	DCAMKL3	88	1000
CHMFL-BTK-11	DDR1	100	1000
CHMFL-BTK-11	DDR2	99	1000
CHMFL-BTK-11	DLK	100	1000
CHMFL-BTK-11	DMPK	100	1000
CHMFL-BTK-11	DMPK2	99	1000
CHMFL-BTK-11	DRAK1	96	1000
CHMFL-BTK-11	DRAK2	95	1000
CHMFL-BTK-11	DYRK1A	100	1000
CHMFL-BTK-11	DYRK1B	65	1000
CHMFL-BTK-11	DYRK2	55	1000
CHMFL-BTK-11	EGFR	88	1000
CHMFL-BTK-11	EGFR(E746-A750del)	90	1000
CHMFL-BTK-11	EGFR(G719C)	97	1000
CHMFL-BTK-11	EGFR(G719S)	88	1000
CHMFL-BTK-11	EGFR(L747-E749del, A750P)	73	1000
CHMFL-BTK-11	EGFR(L747-S752del, P753S)	86	1000
CHMFL-BTK-11	EGFR(L747-T751del,Sins)	77	1000
CHMFL-BTK-11	EGFR(L858R)	70	1000
CHMFL-BTK-11	EGFR(L858R,T790M)	42	1000
CHMFL-BTK-11	EGFR(L861Q)	71	1000
CHMFL-BTK-11	EGFR(S752-I759del)	58	1000
CHMFL-BTK-11	EGFR(T790M)	58	1000
CHMFL-BTK-11	EIF2AK1	92	1000
CHMFL-BTK-11	EPHA1	100	1000
CHMFL-BTK-11	EPHA2	88	1000

CHMFL-BTK-11	EPHA3	97	1000
CHMFL-BTK-11	EPHA4	100	1000
CHMFL-BTK-11	EPHA5	100	1000
CHMFL-BTK-11	EPHA6	100	1000
CHMFL-BTK-11	EPHA7	97	1000
CHMFL-BTK-11	EPHA8	92	1000
CHMFL-BTK-11	EPHB1	100	1000
CHMFL-BTK-11	EPHB2	89	1000
CHMFL-BTK-11	EPHB3	97	1000
CHMFL-BTK-11	EPHB4	100	1000
CHMFL-BTK-11	EPHB6	87	1000
CHMFL-BTK-11	ERBB2	98	1000
CHMFL-BTK-11	ERBB3	90	1000
CHMFL-BTK-11	ERBB4	91	1000
CHMFL-BTK-11	ERK1	100	1000
CHMFL-BTK-11	ERK2	100	1000
CHMFL-BTK-11	ERK3	100	1000
CHMFL-BTK-11	ERK4	96	1000
CHMFL-BTK-11	ERK5	100	1000
CHMFL-BTK-11	ERK8	99	1000
CHMFL-BTK-11	ERN1	76	1000
CHMFL-BTK-11	FAK	100	1000
CHMFL-BTK-11	FER	98	1000
CHMFL-BTK-11	FES	90	1000
CHMFL-BTK-11	FGFR1	88	1000
CHMFL-BTK-11	FGFR2	88	1000
CHMFL-BTK-11	FGFR3	89	1000
CHMFL-BTK-11	FGFR3(G697C)	100	1000
CHMFL-BTK-11	FGFR4	100	1000
CHMFL-BTK-11	FGR	100	1000
CHMFL-BTK-11	FLT1	88	1000
CHMFL-BTK-11	FLT3	100	1000
CHMFL-BTK-11	FLT3(D835H)	100	1000
CHMFL-BTK-11	FLT3(D835Y)	100	1000
CHMFL-BTK-11	FLT3(ITD)	89	1000
CHMFL-BTK-11	FLT3(K663Q)	100	1000
CHMFL-BTK-11	FLT3(N841I)	100	1000
CHMFL-BTK-11	FLT3(R834Q)	99	1000
CHMFL-BTK-11	FLT3-autoinhibited	100	1000
CHMFL-BTK-11	FLT4	100	1000
CHMFL-BTK-11	FRK	100	1000
CHMFL-BTK-11	FYN	99	1000
CHMFL-BTK-11	GAK	100	1000
CHMFL-BTK-11	GCN2(Kin.Dom.2,S808G)	100	1000
CHMFL-BTK-11	GRK1	71	1000
CHMFL-BTK-11	GRK4	94	1000
CHMFL-BTK-11	GRK7	100	1000
CHMFL-BTK-11	GSK3A	90	1000
CHMFL-BTK-11	GSK3B	100	1000
CHMFL-BTK-11	HASPIN	59	1000
CHMFL-BTK-11	HCK	100	1000
CHMFL-BTK-11	HIPK1	79	1000
CHMFL-BTK-11	HIPK2	96	1000
CHMFL-BTK-11	HIPK3	100	1000
CHMFL-BTK-11	HIPK4	94	1000
CHMFL-BTK-11	HPK1	94	1000
CHMFL-BTK-11	HUNK	86	1000

CHMFL-BTK-11	ICK	100	1000
CHMFL-BTK-11	IGF1R	100	1000
CHMFL-BTK-11	IKK-alpha	96	1000
CHMFL-BTK-11	IKK-beta	86	1000
CHMFL-BTK-11	IKK-epsilon	96	1000
CHMFL-BTK-11	INSR	82	1000
CHMFL-BTK-11	INSRR	100	1000
CHMFL-BTK-11	IRAK1	87	1000
CHMFL-BTK-11	IRAK3	98	1000
CHMFL-BTK-11	IRAK4	82	1000
CHMFL-BTK-11	ITK	100	1000
CHMFL-BTK-11	JAK1(JH1domain-catalytic)	100	1000
CHMFL-BTK-11	JAK1(JH2domain-pseudokinase)	82	1000
CHMFL-BTK-11	JAK2(JH1domain-catalytic)	78	1000
CHMFL-BTK-11	JAK3(JH1domain-catalytic)	5.7	1000
CHMFL-BTK-11	JNK1	72	1000
CHMFL-BTK-11	JNK2	100	1000
CHMFL-BTK-11	JNK3	100	1000
CHMFL-BTK-11	KIT	100	1000
CHMFL-BTK-11	KIT(A829P)	98	1000
CHMFL-BTK-11	KIT(D816H)	100	1000
CHMFL-BTK-11	KIT(D816V)	97	1000
CHMFL-BTK-11	KIT(L576P)	100	1000
CHMFL-BTK-11	KIT(V559D)	100	1000
CHMFL-BTK-11	KIT(V559D,T670I)	99	1000
CHMFL-BTK-11	KIT(V559D,V654A)	100	1000
CHMFL-BTK-11	KIT-autoinhibited	83	1000
CHMFL-BTK-11	LATS1	99	1000
CHMFL-BTK-11	LATS2	100	1000
CHMFL-BTK-11	LCK	100	1000
CHMFL-BTK-11	LIMK1	100	1000
CHMFL-BTK-11	LIMK2	100	1000
CHMFL-BTK-11	LKB1	91	1000
CHMFL-BTK-11	LOK	97	1000
CHMFL-BTK-11	LRRK2	96	1000
CHMFL-BTK-11	LRRK2(G2019S)	95	1000
CHMFL-BTK-11	LTK	100	1000
CHMFL-BTK-11	LYN	94	1000
CHMFL-BTK-11	LZK	100	1000
CHMFL-BTK-11	MAK	100	1000
CHMFL-BTK-11	MAP3K1	100	1000
CHMFL-BTK-11	MAP3K15	80	1000
CHMFL-BTK-11	MAP3K2	99	1000
CHMFL-BTK-11	MAP3K3	100	1000
CHMFL-BTK-11	MAP3K4	97	1000
CHMFL-BTK-11	MAP4K2	98	1000
CHMFL-BTK-11	MAP4K3	100	1000
CHMFL-BTK-11	MAP4K4	97	1000
CHMFL-BTK-11	MAP4K5	100	1000
CHMFL-BTK-11	MAPKAPK2	99	1000
CHMFL-BTK-11	MAPKAPK5	100	1000
CHMFL-BTK-11	MARK1	82	1000
CHMFL-BTK-11	MARK2	92	1000
CHMFL-BTK-11	MARK3	100	1000
CHMFL-BTK-11	MARK4	79	1000
CHMFL-BTK-11	MAST1	100	1000
CHMFL-BTK-11	MEK1	100	1000

CHMFL-BTK-11	MEK2	92	1000
CHMFL-BTK-11	MEK3	86	1000
CHMFL-BTK-11	MEK4	100	1000
CHMFL-BTK-11	MEK5	80	1000
CHMFL-BTK-11	MEK6	79	1000
CHMFL-BTK-11	MELK	73	1000
CHMFL-BTK-11	MERTK	100	1000
CHMFL-BTK-11	MET	100	1000
CHMFL-BTK-11	MET(M1250T)	100	1000
CHMFL-BTK-11	MET(Y1235D)	100	1000
CHMFL-BTK-11	MINK	64	1000
CHMFL-BTK-11	MKK7	77	1000
CHMFL-BTK-11	MKNK1	92	1000
CHMFL-BTK-11	MKNK2	98	1000
CHMFL-BTK-11	MLCK	98	1000
CHMFL-BTK-11	MLK1	100	1000
CHMFL-BTK-11	MLK2	94	1000
CHMFL-BTK-11	MLK3	100	1000
CHMFL-BTK-11	MRCKA	100	1000
CHMFL-BTK-11	MRCKB	100	1000
CHMFL-BTK-11	MST1	100	1000
CHMFL-BTK-11	MST1R	98	1000
CHMFL-BTK-11	MST2	97	1000
CHMFL-BTK-11	MST3	100	1000
CHMFL-BTK-11	MST4	96	1000
CHMFL-BTK-11	MTOR	62	1000
CHMFL-BTK-11	MUSK	100	1000
CHMFL-BTK-11	MYLK	82	1000
CHMFL-BTK-11	MYLK2	100	1000
CHMFL-BTK-11	MYLK4	100	1000
CHMFL-BTK-11	MYO3A	100	1000
CHMFL-BTK-11	MYO3B	90	1000
CHMFL-BTK-11	NDR1	68	1000
CHMFL-BTK-11	NDR2	99	1000
CHMFL-BTK-11	NEK1	82	1000
CHMFL-BTK-11	NEK10	97	1000
CHMFL-BTK-11	NEK11	96	1000
CHMFL-BTK-11	NEK2	100	1000
CHMFL-BTK-11	NEK3	87	1000
CHMFL-BTK-11	NEK4	100	1000
CHMFL-BTK-11	NEK5	73	1000
CHMFL-BTK-11	NEK6	100	1000
CHMFL-BTK-11	NEK7	100	1000
CHMFL-BTK-11	NEK9	100	1000
CHMFL-BTK-11	NIK	100	1000
CHMFL-BTK-11	NIM1	100	1000
CHMFL-BTK-11	NLK	100	1000
CHMFL-BTK-11	OSR1	96	1000
CHMFL-BTK-11	p38-alpha	76	1000
CHMFL-BTK-11	p38-beta	100	1000
CHMFL-BTK-11	p38-delta	96	1000
CHMFL-BTK-11	p38-gamma	79	1000
CHMFL-BTK-11	PAK1	100	1000
CHMFL-BTK-11	PAK2	99	1000
CHMFL-BTK-11	PAK3	94	1000
CHMFL-BTK-11	PAK4	95	1000
CHMFL-BTK-11	PAK6	100	1000

CHMFL-BTK-11	PAK7	100	1000
CHMFL-BTK-11	PCTK1	100	1000
CHMFL-BTK-11	PCTK2	94	1000
CHMFL-BTK-11	PCTK3	90	1000
CHMFL-BTK-11	PDGFRA	100	1000
CHMFL-BTK-11	PDGFRB	100	1000
CHMFL-BTK-11	PDPK1	90	1000
CHMFL-BTK-11	PFCDPK1(P.falciparum)	97	1000
CHMFL-BTK-11	PFPK5(P.falciparum)	100	1000
CHMFL-BTK-11	PFTAIRE2	100	1000
CHMFL-BTK-11	PFTK1	100	1000
CHMFL-BTK-11	PHKG1	100	1000
CHMFL-BTK-11	PHKG2	100	1000
CHMFL-BTK-11	PIK3C2B	100	1000
CHMFL-BTK-11	PIK3C2G	95	1000
CHMFL-BTK-11	PIK3CA	100	1000
CHMFL-BTK-11	PIK3CA(C420R)	74	1000
CHMFL-BTK-11	PIK3CA(E542K)	91	1000
CHMFL-BTK-11	PIK3CA(E545A)	76	1000
CHMFL-BTK-11	PIK3CA(E545K)	69	1000
CHMFL-BTK-11	PIK3CA(H1047L)	85	1000
CHMFL-BTK-11	PIK3CA(H1047Y)	82	1000
CHMFL-BTK-11	PIK3CA(I800L)	62	1000
CHMFL-BTK-11	PIK3CA(M1043I)	99	1000
CHMFL-BTK-11	PIK3CA(Q546K)	97	1000
CHMFL-BTK-11	PIK3CB	100	1000
CHMFL-BTK-11	PIK3CD	100	1000
CHMFL-BTK-11	PIK3CG	73	1000
CHMFL-BTK-11	PIK4CB	100	1000
CHMFL-BTK-11	PIM1	82	1000
CHMFL-BTK-11	PIM2	100	1000
CHMFL-BTK-11	PIM3	95	1000
CHMFL-BTK-11	PIP5K1A	100	1000
CHMFL-BTK-11	PIP5K1C	82	1000
CHMFL-BTK-11	PIP5K2B	100	1000
CHMFL-BTK-11	PIP5K2C	90	1000
CHMFL-BTK-11	PKAC-alpha	98	1000
CHMFL-BTK-11	PKAC-beta	100	1000
CHMFL-BTK-11	PKMYT1	94	1000
CHMFL-BTK-11	PKN1	100	1000
CHMFL-BTK-11	PKN2	100	1000
CHMFL-BTK-11	PKNB(M.tuberculosis)	69	1000
CHMFL-BTK-11	PLK1	99	1000
CHMFL-BTK-11	PLK2	91	1000
CHMFL-BTK-11	PLK3	100	1000
CHMFL-BTK-11	PLK4	56	1000
CHMFL-BTK-11	PRKCD	86	1000
CHMFL-BTK-11	PRKCE	97	1000
CHMFL-BTK-11	PRKCH	84	1000
CHMFL-BTK-11	PRKCI	69	1000
CHMFL-BTK-11	PRKCQ	100	1000
CHMFL-BTK-11	PRKD1	94	1000
CHMFL-BTK-11	PRKD2	100	1000
CHMFL-BTK-11	PRKD3	100	1000
CHMFL-BTK-11	PRKG1	100	1000
CHMFL-BTK-11	PRKG2	98	1000
CHMFL-BTK-11	PRKR	80	1000

CHMFL-BTK-11	PRKX	100	1000
CHMFL-BTK-11	PRP4	93	1000
CHMFL-BTK-11	PYK2	100	1000
CHMFL-BTK-11	QSK	92	1000
CHMFL-BTK-11	RAF1	100	1000
CHMFL-BTK-11	RET	100	1000
CHMFL-BTK-11	RET(M918T)	100	1000
CHMFL-BTK-11	RET(V804L)	97	1000
CHMFL-BTK-11	RET(V804M)	100	1000
CHMFL-BTK-11	RIOK1	66	1000
CHMFL-BTK-11	RIOK2	87	1000
CHMFL-BTK-11	RIOK3	80	1000
CHMFL-BTK-11	RIPK1	90	1000
CHMFL-BTK-11	RIPK2	100	1000
CHMFL-BTK-11	RIPK4	100	1000
CHMFL-BTK-11	RIPK5	99	1000
CHMFL-BTK-11	ROCK1	89	1000
CHMFL-BTK-11	ROCK2	97	1000
CHMFL-BTK-11	ROS1	94	1000
CHMFL-BTK-11	RPS6KA4(Kin.Dom.1-N-terminal)	100	1000
CHMFL-BTK-11	RPS6KA4(Kin.Dom.2-C-terminal)	100	1000
CHMFL-BTK-11	RPS6KA5(Kin.Dom.1-N-terminal)	94	1000
CHMFL-BTK-11	RPS6KA5(Kin.Dom.2-C-terminal)	89	1000
CHMFL-BTK-11	RSK1(Kin.Dom.1-N-terminal)	82	1000
CHMFL-BTK-11	RSK1(Kin.Dom.2-C-terminal)	100	1000
CHMFL-BTK-11	RSK2(Kin.Dom.1-N-terminal)	85	1000
CHMFL-BTK-11	RSK2(Kin.Dom.2-C-terminal)	100	1000
CHMFL-BTK-11	RSK3(Kin.Dom.1-N-terminal)	100	1000
CHMFL-BTK-11	RSK3(Kin.Dom.2-C-terminal)	100	1000
CHMFL-BTK-11	RSK4(Kin.Dom.1-N-terminal)	91	1000
CHMFL-BTK-11	RSK4(Kin.Dom.2-C-terminal)	100	1000
CHMFL-BTK-11	S6K1	100	1000
CHMFL-BTK-11	SBK1	81	1000
CHMFL-BTK-11	SGK	65	1000
CHMFL-BTK-11	SgK110	90	1000
CHMFL-BTK-11	SGK2	85	1000
CHMFL-BTK-11	SGK3	86	1000
CHMFL-BTK-11	SIK	100	1000
CHMFL-BTK-11	SIK2	98	1000
CHMFL-BTK-11	SLK	100	1000
CHMFL-BTK-11	SNARK	90	1000
CHMFL-BTK-11	SNRK	94	1000
CHMFL-BTK-11	SRC	98	1000
CHMFL-BTK-11	SRMS	88	1000
CHMFL-BTK-11	SRPK1	100	1000
CHMFL-BTK-11	SRPK2	100	1000
CHMFL-BTK-11	SRPK3	96	1000
CHMFL-BTK-11	STK16	91	1000
CHMFL-BTK-11	STK33	89	1000
CHMFL-BTK-11	STK35	100	1000
CHMFL-BTK-11	STK36	97	1000
CHMFL-BTK-11	STK39	72	1000
CHMFL-BTK-11	SYK	93	1000
CHMFL-BTK-11	TAK1	98	1000
CHMFL-BTK-11	TAOK1	100	1000
CHMFL-BTK-11	TAOK2	91	1000
CHMFL-BTK-11	TAOK3	93	1000

CHMFL-BTK-11	TBK1	81	1000
CHMFL-BTK-11	TEC	56	1000
CHMFL-BTK-11	TESK1	97	1000
CHMFL-BTK-11	TGFBR1	100	1000
CHMFL-BTK-11	TGFBR2	93	1000
CHMFL-BTK-11	TIE1	100	1000
CHMFL-BTK-11	TIE2	100	1000
CHMFL-BTK-11	TLK1	100	1000
CHMFL-BTK-11	TLK2	78	1000
CHMFL-BTK-11	TNIK	99	1000
CHMFL-BTK-11	TNK1	100	1000
CHMFL-BTK-11	TNK2	100	1000
CHMFL-BTK-11	TNNI3K	100	1000
CHMFL-BTK-11	TRKA	97	1000
CHMFL-BTK-11	TRKB	100	1000
CHMFL-BTK-11	TRKC	93	1000
CHMFL-BTK-11	TRPM6	100	1000
CHMFL-BTK-11	TSSK1B	100	1000
CHMFL-BTK-11	TTK	100	1000
CHMFL-BTK-11	TXK	51	1000
CHMFL-BTK-11	TYK2(JH1domain-catalytic)	92	1000
CHMFL-BTK-11	TYK2(JH2domain-pseudokinase)	97	1000
CHMFL-BTK-11	TYRO3	88	1000
CHMFL-BTK-11	ULK1	100	1000
CHMFL-BTK-11	ULK2	100	1000
CHMFL-BTK-11	ULK3	86	1000
CHMFL-BTK-11	VEGFR2	100	1000
CHMFL-BTK-11	VRK2	95	1000
CHMFL-BTK-11	WEE1	100	1000
CHMFL-BTK-11	WEE2	100	1000
CHMFL-BTK-11	WNK1	93	1000
CHMFL-BTK-11	WNK3	100	1000
CHMFL-BTK-11	YANK1	80	1000
CHMFL-BTK-11	YANK2	91	1000
CHMFL-BTK-11	YANK3	100	1000
CHMFL-BTK-11	YES	100	1000
CHMFL-BTK-11	YSK1	79	1000
CHMFL-BTK-11	YSK4	97	1000
CHMFL-BTK-11	ZAK	79	1000
CHMFL-BTK-11	ZAP70	100	1000

Supplemental Table 2 The standards of arthritis global assessment* of AA rats

	**0**	**1**	**2**
**ear**	no nodules	"arthritis" nodules in one ear	"arthritis" nodules in two ears
**nose**	no connective tissue swelling	connective tissue swelling	-
**tail**	no nodules	"arthritis" nodules	-
**front paw**	no swelling	a front paw swelling	two front paw swelling
**hind paw**	no swelling	a hind paw swelling	two hind paw swelling

*Each rat is awarded 8 points at most.

Supplemental Materials

Supplemental Table 3 The evaluation standards of arthritis index* of AA rats

points	Symptoms
**0**	normal
**1**	Erythema and slight swelling of ankle
**2**	Erythema and slight swelling of ankle and metatarsophalangeal joints (or metacarpal joints)
**3**	Erythema and moderate swelling of ankle and metatarsophalangeal joints (or metacarpal joints)
**4**	Erythema and severe swelling of ankle and metatarsophalangeal joints

*Each rat has 4 paw, and is awarded 16 points at most.

Supplemental Table 4 The evaluation standards of quantification of H&E staining

points	Symptoms
**0**	normal ankle joint
**1**	occasional synovial hyperplasia
**2**	definite arthritis with a few layers of flat to rounded synovial lining
**3**	clear hyperplasia of the synovium with three or more layers of loosely arranged lining cells
**4**	severe synovitis with erosions of articular cartilages and subchondral bones
